# Supplementary material for: Unveiling the Bioactive Potential of the Invasive Jellyfish Phyllorhiza punctata Through Integrative Transcriptomic and Proteomic Analyses
Source: Biomolecules. 2025 Aug 4;15(8):1121. doi: 10.3390/biom15081121 (PMC12383608; doi:10.3390/biom15081121)
Supplement: Supplementary file 1 [file biomolecules-15-01121-s001.zip › Suplementary Material/FileS3/PEPFOLD4/XP_065053669_5752/clusters.html]

 PEP-FOLD clusters


Download archive of all models  here   

|  |  |  |
| --- | --- | --- |
| # | file | sOPEP |
| # cluster 1 - Size: 96 |  |  |
|  | XP\_065053669\_5752-model1 | -21.9703 |
|  | XP\_065053669\_5752-model1.1 | -21.9703 |
|  | XP\_065053669\_5752-model1.2 | -21.9703 |
|  | XP\_065053669\_5752-model1.3 | -21.9703 |
|  | XP\_065053669\_5752-model1.4 | -21.9703 |
|  | XP\_065053669\_5752-model1.5 | -21.9703 |
|  | XP\_065053669\_5752-model1.6 | -21.9703 |
|  | XP\_065053669\_5752-model1.7 | -21.9703 |
|  | XP\_065053669\_5752-model1.8 | -21.9703 |
|  | XP\_065053669\_5752-model1.9 | -21.9703 |
|  | XP\_065053669\_5752-model1.10 | -21.9703 |
|  | XP\_065053669\_5752-model1.11 | -21.9703 |
|  | XP\_065053669\_5752-model1.12 | -21.9703 |
|  | XP\_065053669\_5752-model1.13 | -21.9703 |
|  | XP\_065053669\_5752-model1.14 | -21.9703 |
|  | XP\_065053669\_5752-model1.15 | -21.9703 |
|  | XP\_065053669\_5752-model1.16 | -21.9703 |
|  | XP\_065053669\_5752-model1.17 | -21.9073 |
|  | XP\_065053669\_5752-model1.18 | -21.9073 |
|  | XP\_065053669\_5752-model1.19 | -21.9073 |
|  | XP\_065053669\_5752-model1.20 | -21.9073 |
|  | XP\_065053669\_5752-model1.21 | -21.9073 |
|  | XP\_065053669\_5752-model1.22 | -21.9073 |
|  | XP\_065053669\_5752-model1.23 | -21.9073 |
|  | XP\_065053669\_5752-model1.24 | -21.9073 |
|  | XP\_065053669\_5752-model1.25 | -21.9073 |
|  | XP\_065053669\_5752-model1.26 | -21.9013 |
|  | XP\_065053669\_5752-model1.27 | -21.9013 |
|  | XP\_065053669\_5752-model1.28 | -21.9013 |
|  | XP\_065053669\_5752-model1.29 | -21.9013 |
|  | XP\_065053669\_5752-model1.30 | -21.9013 |
|  | XP\_065053669\_5752-model1.31 | -21.9013 |
|  | XP\_065053669\_5752-model1.32 | -21.9013 |
|  | XP\_065053669\_5752-model1.33 | -21.9013 |
|  | XP\_065053669\_5752-model1.34 | -21.9013 |
|  | XP\_065053669\_5752-model1.35 | -21.9013 |
|  | XP\_065053669\_5752-model1.36 | -21.9013 |
|  | XP\_065053669\_5752-model1.37 | -21.9013 |
|  | XP\_065053669\_5752-model1.38 | -21.9013 |
|  | XP\_065053669\_5752-model1.39 | -21.9013 |
|  | XP\_065053669\_5752-model1.40 | -21.9013 |
|  | XP\_065053669\_5752-model1.41 | -21.9013 |
|  | XP\_065053669\_5752-model1.42 | -21.9013 |
|  | XP\_065053669\_5752-model1.43 | -21.9013 |
|  | XP\_065053669\_5752-model1.44 | -21.9013 |
|  | XP\_065053669\_5752-model1.45 | -21.9013 |
|  | XP\_065053669\_5752-model1.46 | -21.9013 |
|  | XP\_065053669\_5752-model1.47 | -21.8911 |
|  | XP\_065053669\_5752-model1.48 | -21.8687 |
|  | XP\_065053669\_5752-model1.49 | -21.8687 |
|  | XP\_065053669\_5752-model1.50 | -21.8687 |
|  | XP\_065053669\_5752-model1.51 | -21.8101 |
|  | XP\_065053669\_5752-model1.52 | -21.8101 |
|  | XP\_065053669\_5752-model1.53 | -21.8101 |
|  | XP\_065053669\_5752-model1.54 | -21.8101 |
|  | XP\_065053669\_5752-model1.55 | -21.7852 |
|  | XP\_065053669\_5752-model1.56 | -21.7852 |
|  | XP\_065053669\_5752-model1.57 | -21.7852 |
|  | XP\_065053669\_5752-model1.58 | -21.7852 |
|  | XP\_065053669\_5752-model1.59 | -21.7852 |
|  | XP\_065053669\_5752-model1.60 | -21.7846 |
|  | XP\_065053669\_5752-model1.61 | -21.7671 |
|  | XP\_065053669\_5752-model1.62 | -21.7671 |
|  | XP\_065053669\_5752-model1.63 | -21.7491 |
|  | XP\_065053669\_5752-model1.64 | -21.7395 |
|  | XP\_065053669\_5752-model1.65 | -21.7395 |
|  | XP\_065053669\_5752-model1.66 | -21.7395 |
|  | XP\_065053669\_5752-model1.67 | -21.7395 |
|  | XP\_065053669\_5752-model1.68 | -21.7395 |
|  | XP\_065053669\_5752-model1.69 | -21.7395 |
|  | XP\_065053669\_5752-model1.70 | -21.7286 |
|  | XP\_065053669\_5752-model1.71 | -21.7286 |
|  | XP\_065053669\_5752-model1.72 | -21.7253 |
|  | XP\_065053669\_5752-model1.73 | -21.7181 |
|  | XP\_065053669\_5752-model1.74 | -21.7065 |
|  | XP\_065053669\_5752-model1.75 | -21.7065 |
|  | XP\_065053669\_5752-model1.76 | -21.7057 |
|  | XP\_065053669\_5752-model1.77 | -21.7057 |
|  | XP\_065053669\_5752-model1.78 | -21.7057 |
|  | XP\_065053669\_5752-model1.79 | -21.7057 |
|  | XP\_065053669\_5752-model1.80 | -21.7057 |
|  | XP\_065053669\_5752-model1.81 | -21.7024 |
|  | XP\_065053669\_5752-model1.82 | -21.7024 |
|  | XP\_065053669\_5752-model1.83 | -21.6641 |
|  | XP\_065053669\_5752-model1.84 | -21.6369 |
|  | XP\_065053669\_5752-model1.85 | -21.623 |
|  | XP\_065053669\_5752-model1.86 | -21.623 |
|  | XP\_065053669\_5752-model1.87 | -21.623 |
|  | XP\_065053669\_5752-model1.88 | -21.623 |
|  | XP\_065053669\_5752-model1.89 | -21.603 |
|  | XP\_065053669\_5752-model1.90 | -21.5994 |
|  | XP\_065053669\_5752-model1.91 | -21.5959 |
|  | XP\_065053669\_5752-model1.92 | -21.5959 |
|  | XP\_065053669\_5752-model1.93 | -21.5728 |
|  | XP\_065053669\_5752-model1.94 | -21.4866 |
|  | XP\_065053669\_5752-model1.95 | -21.3956 |
| # cluster 2 - Size: 3 |  |  |
|  | XP\_065053669\_5752-model2 | -15.1779 |
|  | XP\_065053669\_5752-model2.1 | -15.1405 |
|  | XP\_065053669\_5752-model2.2 | -14.6092 |
| # cluster 3 - Size: 1 |  |  |
|  | XP\_065053669\_5752-model3 | -14.7362 |
|  |  |  |
